# Efficacy of serial pancreatic juice aspiration cytological examination for focal pancreatic duct stenosis: Multicenter, retrospective, cohort study

**DOI:** 10.1055/a-2655-6348

**Published:** 2025-07-29

**Authors:** Yosuke Ohashi, Takuji Iwashita, Shota Iwata, Akihiko Senju, Shinya Uemura, Keisuke Iwata, Akinori Maruta, Junji Kawaguchi, Naoki Mita, Masatoshi Mabuchi, Yasuhiro Oshima, Masahito Shimizu

**Affiliations:** 1 476117 First Department of Internal Medicine, Gifu University Hospital, Gifu, 501-1194, Japan; 2 38226 Gastroenterology, Gifu Municipal Hospital, Gifu, Japan; 3 68266 Gastroenterology, Gifu Prefectural General Medical Center, Noisshiki 4-6-1, Gifu city, Japan; 4 73505 Department of Gastroenterology, Matsunami General Hospital, Gifu, Japan; 5 157624 Department of Gastroenterology, Chuno Kosei Hospital, Gifu, Japan; 6 469489 Department of Gastroenterology, JA Gifu Kouseiren Seino Kosei Hospital, Gifu, Japan; 7 73504 Department of Gastroenterology, Central Japan International Medical Center, Gifu, Japan

**Keywords:** Pancreatobiliary (ERCP/PTCD), GI Pathology, Strictures

## Abstract

**Background and study aims:**

Early diagnosis of pancreatic cancer is crucial for improving patient prognosis. However, diagnosing pancreatic cancer in the absence of a distinct mass is challenging due to limitations of endoscopic ultrasound-guided fine-needle aspiration (EUS-FNA). Recent studies have suggested that serial pancreatic juice aspiration cytological examination (SPACE) for focal pancreatic duct stenosis may improve diagnostic yield in cases of pancreatic cancer without obvious mass. The aim of this study was to evaluate efficacy of SPACE in diagnosis of focal pancreatic duct stenosis.

**Patients and methods:**

A retrospective analysis of data from patients who underwent SPACE for pancreatic ductal stenosis between January 2017 and January 2023 was performed. Primary outcomes included diagnostic capability (sensitivity, specificity, and accuracy) and safety of SPACE.

**Results:**

SPACE was performed on 46 patients with focal pancreatic duct stenosis. Initial cytology demonstrated sensitivity of 45.0%, specificity of 84.6%, and accuracy of 67.3%. In contrast, SPACE yielded a significantly improved sensitivity of 90.0% ( *P* = 0.006) and an accuracy of 89.1% ( *P* = 0.045). Sensitivity of SPACE increased with the number of cytological examinations: 45% for one submission; 70% for two; 80% for three; 85% for four; and plateaued at 90% for five or more 5. Adverse events occurred in 19.6% of patients, including pancreatitis (mild in 7, moderate in 1) and one case of guidewire penetration of the pancreatic duct.

**Conclusions:**

SPACE was a valuable method for obtaining pathological specimens of focal pancreatic duct stenosis. It yielded improved diagnostic sensitivity and accuracy compared with initial cytology alone.

## Introduction

In 2020, 495,000 new cases of pancreatic cancer and 466,000 related deaths were reported worldwide. Incidence and mortality rates for pancreatic cancer are increasing worldwide, both of which underscore the poor prognosis of the disease 1 . In Japan, pancreatic cancer is known to have a poor prognosis, with an overall 5-year survival rate of only 8.5% 2 , with a similarly poor prognosis in the United States, with a 5-year rate of approximately 10% 3 . One explanation for the poor prognosis of pancreatic cancer is that an advanced local or metastatic state at diagnosis often precludes surgery, which is the only curative treatment. Moreover, lack of effective chemotherapy for advanced pancreatic cancer also contributes to poor prognosis. However, 5-year survival rates for patients undergoing surgical resection at Union for International Cancer Control (UICC) stage 0 (high-grade pancreatic intraepithelial neoplasia [PanIN] or pancreatic carcinoma in situ [CIS]) and stage 1A (tumors confined to the pancreatic duct or measuring ≤ 20 mm) are 85.8% and 68.7%, respectively 4 . Therefore, identifying and diagnosing pancreatic cancer in its early stage(s) is important for improving prognosis.

In diagnosis of early-stage pancreatic cancer, various indirect findings, which do not directly detect pancreatic cancer but are associated with the tumor, play an important role. The most important indirect imaging findings suggesting early-stage pancreatic cancer include pancreatic duct stenosis and dilation of the main pancreatic duct at its tail side because identifying a tiny tumor through imaging and performing endoscopic ultrasound (EUS)-guided fine-needle aspiration (EUS-FNA) for definitive diagnosis remains challenging. To obtain pathological samples for evaluation of early-stage pancreatic cancer, pancreatic juice cytology and pancreatic duct brush cytology have been used as endoscopic retrograde cholangiopancreatography (ERCP)-related procedures. Gene and microRNA analyses of pancreatic juice obtained from an endoscopic nasopancreatic drainage (ENPD) catheter also may be effective 5 . However, challenges persist, including low diagnostic sensitivity and occurrence of adverse events (AEs), such as post-ERCP pancreatitis. Recently, serial pancreatic juice aspiration cytological examination (SPACE), a technique used to collect pancreatic juice multiple times through an ENPD catheter, has demonstrated promise for improving sensitivity; however, studies addressing SPACE are limited 6 7 8 . As such, the present investigation aimed to evaluate utility and safety of SPACE for diagnosing focal pancreatic duct stenosis.

## Patients and methods

### Study design and patient selection

**Fig. 1:**
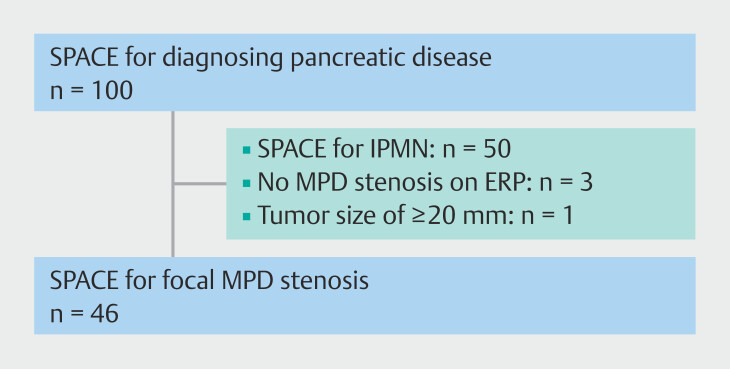
Flow-diagram illustrating participant enrollment process. ERP, endoscopic retrograde pancreatography; IPMN, intraductal papillary mucinous neoplasm; MPD, main pancreatic duct; SPACE, serial pancreatic aspiration cytological examination.

This multicenter, retrospective cohort study was conducted at Gifu University Hospital, Gifu Municipal Hospital, Gifu Prefectural General Medical Center (Gifu), Matsunami General Hospital (Kasamatsu), Chuno Kosei Hospital (Seki), Seino Kosei Hospital (Ibi), and the Central Japan International Medical Center (Minokamo). Information housed in a database that included all ERCP procedures performed at these facilities between January 2017 and January 2023 was analyzed. Eligibility criteria were as follows: underwent SPACE for focal pancreatic duct stenosis and imaging tests (computed tomography [CT], magnetic resonance imaging [MRI], and EUS) revealing no mass or small lesion measuring < 20 mm in which EUS-FNA was difficult or negative. Patients in whom SPACE was performed to evaluate pancreatic cystic disease, including intraductal papillary mucinous neoplasm (IPMN), and those in whom pancreatic tumors measured > 20 mm in size were excluded ( Fig. 1 ). All patients provided informed written consent to participate. The study was conducted in accordance with the human and ethical principles of research set forth in the Helsinki Declaration. The study protocol was approved by the Institutional Review Board of Gifu University Hospital and included all participating centers.

### SPACE

**Fig. 2:**
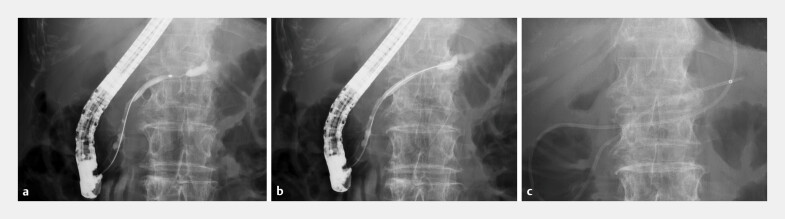
Serial pancreatic aspiration cytological examination. **a** Insertion of the canulation catheter into the pancreatic duct and performance of main pancreatography. **b** Placement of the guidewire at the tail side of the MPD. **c** Placement of a 4F to 6F ENPD catheter in the tail side of the MPD to the pancreatic duct stenosis. ENPD, endoscopic nasopancreatic drainage; MPD, main pancreatic duct.

**Fig. 3:**
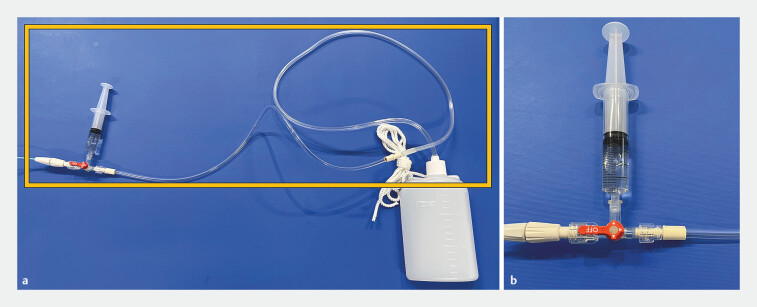
Collection of pancreatic juice. **a** T-shaped stopcock and extension tube are connected between the bottle and the ENPD catheter. The fluid in the catheter is collected, excluding that in the bottle. **b** Fresh pancreatic juice is collected from a T-shaped stopcock. ENPD, endoscopic nasopancreatic drainage.

ERCP was performed using a duodenoscope (TJF-Q290V, TJF-260V, or JF-260V; Olympus, Tokyo, Japan) and pancreatic duct cannulation was performed using an ERCP catheter. A 0.025-inch guidewire was inserted into the main pancreatic duct through the stenosis and the pancreatic duct was evaluated using contrast injection. An ENPD catheter (EN-4A11–260S11 or EN-5A14–260S12, Gadelius Medical, Tokyo, Japan; G21466 Nasal Pancreatic Drainage Set, Cook Medical, Bloomington, Indiana, United States; PBD-V811W, Olympus, Tokyo, Japan) was placed across the stenosis ( Fig. 2 ). In cases in which the guidewire or catheter could not pass through the stenosis, the catheter was placed immediately before the stenosis. Specimens were collected on the day of catheter placement. Samples were collected from the ENPD catheter at 1- to 6-hour intervals, with multiple collections throughout the day. The pancreatic juice collected in the bottle was discarded. A three-way stopcock and extension tube were connected between the ENPD catheter and bottle, enabling approximately 2 mL of fresh pancreatic juice to be collected from the tube ( Fig. 3 ). In all participating hospitals, pancreatic juice samples collected via the ENPD catheter were centrifuged and smears were prepared from the resulting cell pellets on glass slides. Cytological evaluation was primarily performed using Papanicolaou staining, with May-Giemsa or PAS staining occasionally applied as supplementary methods when considered necessary. All specimens were evaluated by experienced pathologists following staining.

### Study outcomes and definitions

The primary outcome was comparison of diagnostic capability of initial cytology and SPACE for pancreatic cancer. Secondary outcomes included diagnostic sensitivity according to the number of cytological samples subjected to SPACE and SPACE-related AEs. Pancreatic juice specimens were initially evaluated by an experienced pathologist to assess their suitability for cytological analysis. Samples were considered adequate if they contained a sufficient number of pancreatic ductal epithelial cells with well-preserved morphology, allowing for reliable cytological interpretation. After that, pancreatic juice cytological results were categorized as normal, atypical, suspicious, or malignant. Suspicion was strongly suggestive of malignancy. In this study, “suspicious” and “malignant” were defined as positive for malignancy 9 .

Final diagnosis was obtained using one of the following: surgical diagnosis using resected specimens or the results of SPACE, blood tests, or imaging findings with a clinical follow-up period ≥ 12 months. In the final diagnoses, high-grade PanIN, intraductal papillary mucinous adenocarcinoma (IPMC), and pancreatic ductal adenocarcinoma (PDAC) were considered to be malignancies, whereas other lesions were considered to be benign. ERCP-related complications were evaluated in accordance with the American Society for Gastrointestinal Endoscopy workshop report 10 .

Physical examination and laboratory investigations, including serum amylase, were routinely performed the day after the examination, and computed tomography was performed, if necessary, to diagnose post-ERCP pancreatitis. Severity of pancreatitis was evaluated using Cotton’s classification 11 .

### Statistical analysis

Continuous variables are expressed as median with range and were compared using the Wilcoxon rank-sum test. Categorical variables were compared using the Fisher’s exact test. For diagnostic results, sensitivity, specificity, positive predictive value (PPV), negative predictive value (NPV), and accuracy were calculated with corresponding 95% confidence interval (CI). Differences with *P* < 0.05 were considered to be statistically significant. All statistical analyses were performed using EZR (Saitama Medical Center, Jichi Medical University, Saitama, Japan), a graphical user interface for R (R Foundation for Statistical Computing, Vienna, Austria) 12 .

## Results

### Basic characteristics

The study included data from 46 patients with a median age of 76.5 years (range, 39–91 years), of whom 27 (58.9%) were male. Median follow-up period for all patients was 33 months (range, 3–73 months). Median blood biochemistry values were as follows: amylase, 95 U/L (range, 43–982 U/L); carcinoembryonic antigen (CEA), 2.5 ng/mL (range, 0.8–9.4 ng/mL); and carbohydrate antigen 19–9 (CA19–9), 12.7 U/mL (range, 0.4–1024 U/mL). Median diameter of the main pancreatic duct at the tail side was 5.2 mm (range, 2.5–9.8 mm). EUS revealed nodules in the stenosis in 16 patients (34.8%). Median diameter of the nodules was 9 mm (range, 2–14.9 mm). EUS-FNA was performed in seven patients (15.2%), all of whom were negative for malignancy. The main pancreatic duct stenosis was located in the head in 18 patients (39.1%), the body in 19 (41.3%), and the tail in nine (19.6%) ( Table 1 ). Final diagnosis in 19 of 46 patients was based on surgical specimens and, in the remaining 27, was based on clinical course with results of SPACE and imaging studies. Final diagnoses were malignancy in 20 patients (PDAC [n = 15]; high-grade PanIn [n = 3]; and IPMC [n = 2]) and benign in 26 (benign pancreatic duct stenosis [n = 15]; chronic pancreatitis [n = 5]; intraductal papillary mucinous adenoma [n = 3]; autoimmune pancreatitis [n = 2], and low-grade PanIn [n = 1]) ( Table 2 ).

**Table:** **Table 1** Patient characteristics.

	All patients (n = 46)
Age, median (range)	76.5 (39–91)
Male, n (%)	27 (58.9)
Follow-up period, months, median (range)	33 (3–73)
Amylase, U/L, median (range)	95 (43–982)
CEA, ng/mL, median (range)	2.5 (0.8–9.4)
CA19–9, U/mL, median (range)	12.7 (0.4–1024)
MPD diameter on the tail side of the pancreas (mm), median (range)	5.2 (2.5–9.8)
A mass that can be detected by EUS, n (%)	16 (34.8)
Tumor size, mm, median (range)	8.5 (2–14.9)
Performing EUS-FNA, n (%)	7 (15.2)
MPD stenosis location	
head, n (%)	18 (39.1)
body, n (%)	19 (41.3)
tail, n (%)	9 (19.6)
CEA, carcinoembryonic antigen; CA19–9, carbohydrate antigen 19–9; EUS-FNA, endoscopic ultrasound-guided fine-needle aspiration; MPD, main pancreatic duct.	

**Table:** **Table 2** Final diagnosis.

Malignant cases, n=20	
PDAC, n (%)	15 (32.6)
High-grade PanIN, n (%)	3 (6.5)
Noninvasive IPMC, n (%)	2 (4.3)
Benign cases, n = 26	
Benign pancreatic duct stenosis, n (%)	15 (32.6)
Chronic pancreatitis, n (%)	5 (10.9)
IPMA, n (%)	3 (6.5)
AIP, n (%)	2 (4.3)
Low-grade PanIN, n (%)	1 (2.2)
AIP, autoimmune pancreatitis; IPMA, intraductal pancreatic intraepithelial adenoma; IPMC, intraductal papillary mucinous carcinoma; PanIN, pancreatic intraepithelial neoplasia; PDAC, pancreatic ductal adenocarcinoma.	

### SPACE

**Fig. 4:**
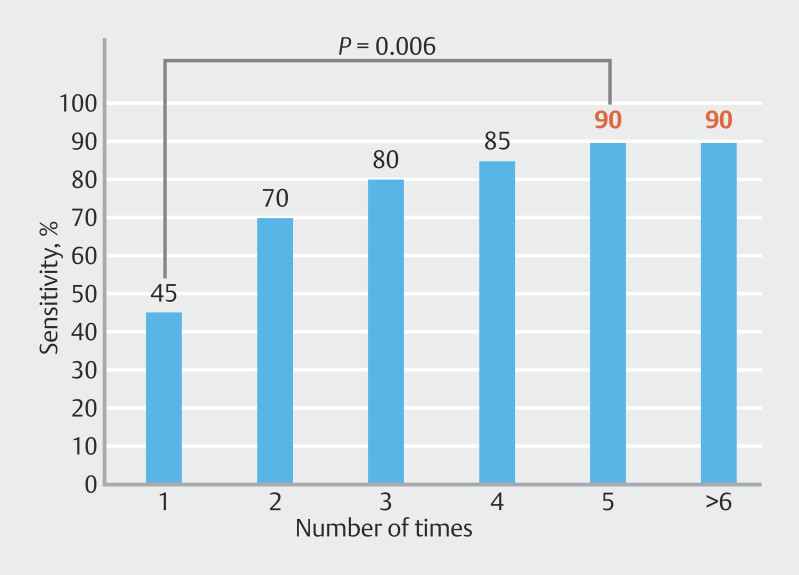
Sensitivity according to number of cytological examinations. Cumulative sensitivity of multiple cytological examinations was calculated and reported.

**Fig. 5:**
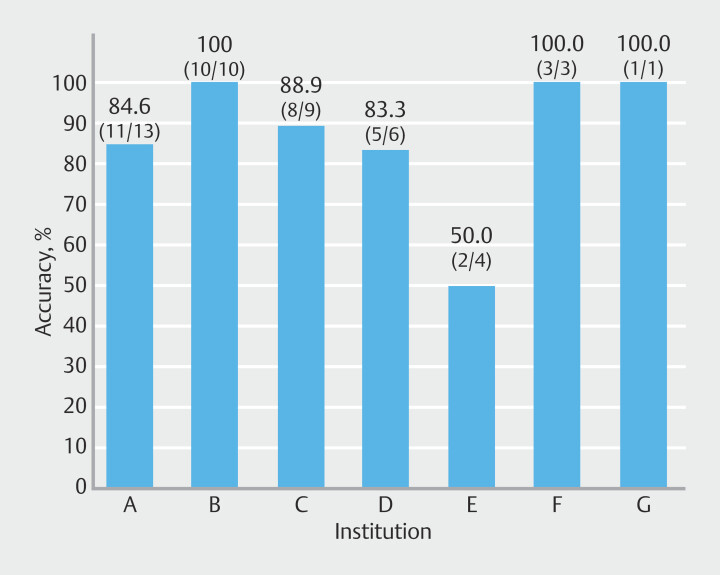
Diagnostic performance of SPACE by institution. Diagnostic accuracy was calculated as the percentage of correct diagnoses out of the total number of cases per institution. SPACE, serial pancreatic aspiration cytological examination.

ENPD catheter placement achieved a success rate of 100% (46/46 patients). ENPD catheters successfully traversed the pancreatic duct stenosis in 43 patients (93.5%). Catheter sizes used were 4F in five patients (10.8%), 5F in 40 (87.0%), and 6F in one (2.2%). Additional procedures performed included biopsy of the stenosis site in three patients (6.5%), brush cytology in 10 (21.7%), and both biopsy and brush cytology in one (2.2%). Median number of cytology submissions per patient was six (range 2–17), with an appropriate cytological sample acquisition rate of 95.4% (248/260) ( Table 3 ). The initial cytology test yielded a sensitivity of 45.0% (9/20 [95% CI 23.1–68.5]), a specificity of 84.6% (22/26 [95% CI 65.1–95.6]), a PPV of 69.2% (9/13 [95% CI 38.6–90.9]), an NPV of 66.7% (22/33 [95% CI 48.2–82.0]), and an accuracy of 67.3% (31/46 [95% CI 52.0–80.5]). In contrast, SPACE yielded a sensitivity of 90.0% (18/20 [95% CI 68.3–98.8]), a specificity of 84.6% (22/26 [95% CI 65.1–95.6]), a PPV of 81.8% (18/22 [95% CI 59.7–94.8]), an NPV of 91.7% (22/24 [95% CI 73.0–99.0]), and an accuracy of 89.1% (40/46 [95% CI 73.7–95.1]). SPACE yielded a significantly higher sensitivity (90% versus [vs.] 45%; *P* = 0.006) and accuracy (89.1% vs. 67.3%; *P* = 0.045) than the initial cytology test ( Table 4 ). Sensitivity of SPACE, based on the number of cytological examinations, increased as the number of submitted cytology samples increased. Diagnostic sensitivity was 45% for one submission, 70% for two, 80% for three, 85% for four, and plateaued at 90% for five or more ( Fig. 4 ). When accuracy was examined for each hospital, the correct diagnosis rate was approximately 80% or higher ( Fig. 5 ).

**Table:** **Table 3** Treatment characteristics.

	All patients (n = 46)
Successful ENPD catheter placement, n (%)	46 (100)
Passing through stenosis by ENPD catheter, n (%)	43 (93.5)
ENPD catheter type	
4F, n (%)	5 (10.8)
5F, n (%)	40 (87.0)
6F, n (%)	1 (2.2)
Combination of SPACE and other examinations	
with MPD biopsy, n (%)	3 (6.5)
with brush cytology, n (%)	10 (21.7)
with MPD biopsy and brush cytology, n (%)	1 (2.2)
Numbers of cytology via ENPD catheter in each patient, median, n (range)	6 (2–17)
Numbers of cytology via ENPD catheter in all patients	260
adequate samples n (%)	248 (95.4)
ENPD, endoscopic nasopancreatic drainage; MPD, main pancreatic duct; SPACE, serial pancreatic aspiration cytological examination.	

**Table:** **Table 4** Comparison of diagnostic ability between initial cytology and SPACE.

	Initial single cytology % (n, 95%CI)	SPACE % (n, 95% CI)	*P* value
Sensitivity	45.0 (9/20, 23.1–68.5)	90.0 (18/20, 68.3–98.8)	0.003
Specificity	84.6 (22/26, 65.1–95.6)	84.6 (22/26, 65.1–95.6)	1
PPV	69.2 (9/13, 38.6–90.9)	81.8 (18/22, 59.7–94.8)	0.433
NPV	66.7 (22/33, 48.2–82.0)	91.7 (22/24, 73.0–99.0)	0.003
Accuracy	67.4 (31/46, 52.0–80.5)	87.0 (40/46, 73.7–95.1)	0.045
CI, confidence interval; NPV, negative predictive value; PPV, positive predictive value; SPACE, serial pancreatic aspiration cytological examination.			

### Safety of SPACE

AEs occurred in 19.6% of patients (9/46), including eight with pancreatitis (mild in 7, moderate in 1), and one with guidewire penetration of the pancreatic duct. All cases of pancreatitis resolved with conservative treatment. A 5F catheter was used for all patients with pancreatitis. The pancreatic duct penetration case involved damage to the normal duct during guidewire passage through the stenosis with contrast leakage through the penetrated area. An NPD was placed proximal to the stenosis and no further events were observed ( Table 5 ).

**Table:** **Table 5** Adverse events of SPACE.

							n=46
**Overall, n (%)**							9 (19.5)
Post ERCP pancreatitis, n (%)							8 (17.4)
Mild/moderate/severe							7/1/0
MPD penetration, n (%)							1 (2.2)
**No**	** Age (years) **	**Gender**	** Pancreatitis grade **	** Amylase (U/mL) **	**ENPD catheter (F)**	**Final diagnosis**	**Adverse events**
1	81	Female	Mild	1029	5	PDAC	PEP
2	75	Female	Mild	551	5	PDAC	PEP
3	91	Female	Mild	947	5	Benign pancreatic duct stenosis	PEP
4	70	Male	Mild	763	5	PDAC	PEP
5	87	Male	Mild	848	5	PDAC	PEP
6	85	Male	Mild	1375	5	Benign pancreatic duct stenosis	PEP
7	83	Female	Mild	804	5	IPMA	PEP
8	71	Female	Moderate	642	5	IPMA	PEP
9	65	Male	-	220	5	Benign pancreatic duct stenosis	MPD penetration
ERCP, endoscopic retrograde cholangiopancreatography; ENPD, endoscopic nasopancreatic drainage; IPMA, intraductal pancreatic intraepithelial adenoma; MPD, main pancreatic duct; PDAC, pancreatic ductal adenocarcinoma.							

## Discussion

The present study evaluated SPACE for diagnosis of indeterminate pancreatic stenosis in cases for which EUS-FNA was challenging. SPACE yielded sensitivity of 90.0%, specificity of 84.6%, and accuracy of 89.1%, demonstrating superior sensitivity and specificity compared with single cytology. Sensitivity of SPACE progressively increased with the number of cytology submissions, reaching a plateau, with a sensitivity of 90% for five or more cytology submissions. AEs occurred in 19.5% of patients (9/46), including post-ERCP pancreatitis in eight and pancreatic duct guidewire penetration in one.

Previous studies have reported that sensitivity of SPACE ranges from 46.7% to 100%, specificity from 83.3% to 100%, and accuracy from 59.8% to 95% ( Table 6 ) 6 7 13 14 15 16 17 18 . In our study, diagnostic capabilities of SPACE for indeterminate focal pancreatic duct stenosis yielded a sensitivity of 90.0%, specificity of 84.6%, and accuracy of 89.1%, which is similar to previous studies. In previous investigations, SPACE was performed on pancreatic masses, focal pancreatic duct stenosis, and IPMN, and a high diagnostic sensitivity (> 75%) has been reported in studies investigating pancreatic duct stenosis; however, few studies have addressed IPMN, demonstrating low sensitivity (as low as 27.3%). It has also been reported that sensitivity of pancreatic juice cytology is better for smaller pancreatic tumors because small pancreatic cancers exhibit stronger intraductal progression than larger lesions, increasing sensitivity of transpapillary collected pancreatic juice cytology 19 . A similar trend was confirmed with SPACE. In addition, SPACE further increases sensitivity by requiring multiple cytological examinations for small pancreatic cancers, although a single submission is effective. A study by Hanada et al 20 . evaluated histological characteristics of surgically resected stage 0 PDAC and corresponding imaging findings, as well as postoperative recurrence of PDAC confined to the remnant pancreas. The authors reported that, in cases of severe pancreatic duct stenosis, the disease did not progress much into the pancreatic parenchyma, but progressed extensively into the pancreatic duct. SPACE may be most effective for small tumors that are difficult to detect using EUS and tumors with intraductal progression, such as pancreatic duct stenosis.

Although multiple cytological examinations certainly increase diagnostic sensitivity, the ideal number of cytological specimen submissions remains unclear and is crucial for balancing efficacy while minimizing the workload of cytopathologists and related costs. Our study evaluated the relationship between sensitivity of pancreatic fluid cytology and number of specimens submitted, revealing that sensitivity was 45% with a single submission and gradually increased with additional submissions, plateauing at 90% after five or more submissions. Nakamura et al 17 . also explored this question by focusing on pancreatic fluid cytology after ENPD placement in patients with suspected resectable pancreatic cancer. They suggested that submitting specimens up to six times could be a reasonable balance between diagnostic yield and considerations of cost, effort for specimen preparation, patient discomfort, and prolonged hospitalization, although there was no exact evidence to support this. Given findings from this study and our previous research, submitting cytology samples six times appears to be a reasonable strategy to maintain high diagnostic accuracy while minimizing cost and effort associated with multiple cytological evaluations.

Reported AEs associated with SPACE include acute pancreatitis and cholangitis, with frequencies ranging from 0% to 13% and 0% to 7.7%, respectively ( Table 6 ) 6 7 13 14 15 16 17 18 . In the present study, AEs were recorded in 19.5% of patients, including eight cases of pancreatitis and one case of pancreatic duct guidewire penetration. Among these AEs, acute pancreatitis warrants attention due to its relatively high frequency of occurrence. Further efforts to reduce incidence of adverse pancreatitis events are necessary to ensure the complete safety of SPACE. A randomized controlled trial by Mouri et al. 21 compared 4F and 5F ENPD catheters to obtain pancreatic juice for cytology in 243 patients. The authors found no significant difference in technical success, with 100% success in both groups and, in the adequacy of pancreatic fluid cytology, with 82% (100/122) in the 4F group and 79.3% (96/121) in the 5F group ( *P* = 0.604). However, incidence of post-ERCP pancreatitis was significantly lower with the 4F catheter: 4.1% (5/122) in the 4F group vs. 12.4% (15/121) in the 5F group ( *P* = 0.021). This finding suggests that use of 4F catheters in pancreatic fluid cytology may be safer, without compromising efficacy. In our study, suitability of a single cytology using 4F and 5F catheters was 100% (29/29) for 4F and 94.6% (212/224) for 5F, with no significant difference ( *P* = 0.37). Incidence of pancreatitis was 0% (0/5 patients) for 4F and 20% (8/40 patients) for 5F. Although the number of cases using 4F was small and not statistically significant ( *P* = 0.57), these findings suggest that use of 4F catheters may reduce AEs without compromising specimen collection performance.

**Table:** **Table 6** Comparison of previous reports on SPACE.

Author/years	Number of cases (n)	Indications	Size of NPD catheter (F)	Number of collections of PC (n)	Sensitivity (%)	Specificity (%)	Accuracy (%)	Post-ERCP pancreatitis (%)	Cholangitis (%)
Iiboshi et al. 2012	27	Focal stenosis and distal dilatation of MPD	5	5.3	100	83.3	95	0	0
Mikata et al. 2013	60	Suspicious PDAC	5 or 6	6	80	100	87	7.5	0
Ikemoto et al. 2021	75	Localized stenosis or distal dilatation of MPD	4 or 5	6	75	NA	NA	7	0
Mie et al. 2022	34	Pancreatic ductal stenosis	5	4	63.6	91.7	73.5	11.8	0
Kitagawa et al. 2022	24	Localized stenosis of main pancreatic duct	5	6	81.8	100	91.7	0	0
Nakamura et al.2023	82	Suspected of having resectable PDAC	4 or 5	6	46.7	95.5	59.8	7.3	0
Satoh et al. 2023	226	Pancreatic masses/suspicious PDAC/IPMN	5 or 6	6	69/78.6/27.3	100/98.1/86.6	78/93.9/75.6	7.3/4.5/13	0/1.5/0.8
Takeda et al. 2023	26	Pancreatic masses	5	6	71.4	100	92.3	7.7	7.7
Our group	46	Pancreatic ductal stenosis	4 or 5 or 6	6	90	84.6	89.1	17.3	0
IPMN, intraductal papillary mucinous neoplasm; MPD, main pancreatic duct; NPD, nasopancreatic drainage; PC, pancreatic juice; PDAC, pancreatic ductal adenocarcinoma; SPACE, serial pancreatic aspiration cytological examination.									

EUS-FNA is generally considered to be the first choice for obtaining a pathological diagnosis of pancreatic tumors due to its high diagnostic accuracy and safety 22 , with a reported sensitivity of 84% to 92%, specificity of 96% to 98%, and accuracy of 86% to 91% 23 24 25 . In addition, EUS-FNA has a low AE rate (0%-2.5%) 26 27 . However, a multicenter study evaluating UICC stage 0 or 1 early-stage pancreatic cancer revealed that EUS-FNA was performed in only 11.8% (6/51) of stage 0 cases, with a sensitivity of 16.7% (1/6). In stage 1 cases, EUS-FNA was performed in 42.3% of patients (63/149), with a sensitivity of 84.3%. Although EUS-FNA demonstrates relatively good diagnostic performance in stage 1 lesions if puncture is possible, its feasibility may be limited considering tumor size. EUS-FNA for stage 0 lesions is challenging to recognize on EUS images due to absence of mass formation. The effectiveness of EUS-FNA in small lesions may also be restricted 28 . SPACE may prove useful for diagnosing CIS and small pancreatic cancers. In our study, of the 46 total cases, 19 patients underwent surgical resection, including 16 malignant and three benign cases. Notably, 11 of 16 malignant cases were early-stage PDAC, classified as stage 0 or 1. These findings suggest that SPACE is effective for early detection of pancreatic cancer ( Table 7 ). Recently, the significance of needle tract seeding (NTS), primarily in the stomach wall, has been highlighted in cases in which distal pancreatectomy is performed after EUS-FNA for pancreatic body or tail cancers 29 30 . SPACE carries no risk for NTS and may be indicated for pancreatic body or tail lesions in which the resection site does not include a puncture site.

**Table:** **Table 7** Summary of surgical resection cases and final diagnoses.

						N = 46
**Overall, n (%)**						19 (41.3)
Malignant cases, n (%)						16 (34.8)
Stage 0/1 cases, n (%)						11 (23.9)
Benign cases, n (%)						3 (6.5)
**Case**	**Age (years)**	**Gender**	**Location**	**SPACE diagnosis**	**Final diagnosis**	**Stage**
1	81	Female	Ph	Malignant	PDAC	IIB
2	64	Female	Ph	Malignant	PDAC	IA
3	82	Female	Pb	Malignant	High-grade PanIN	0
4	75	Female	Pt	Malignant	PDAC	IA
5	65	Male	Ph	Benign	Low-grade PanIN	-
6	59	Female	Pt	Benign	High-grade PanIN	0
7	74	Male	Pt	Benign	PDAC	IA
8	80	Female	Pb	Malignant	High-grade PanIN	0
9	70	Male	Pb	Malignant	PDAC	IA
10	71	Female	Ph	Malignant	IPMA	-
11	82	Male	Pb	Malignant	Noninvasive IPMC	0
12	83	Male	Pb	Malignant	PDAC	IB
13	78	Female	Ph	Malignant	PDAC	IIA
14	67	Male	Ph	Malignant	PDAC	IA
15	85	Female	Pt	Malignant	Noninvasive IPMC	0
16	70	Male	Pb	Malignant	PDAC	IIA
17	63	Female	Pb	Malignant	PDAC	IB
18	70	Female	Ph	Malignant	PDAC	IIB
19	83	Female	Ph	Malignant	IPMA	-
IPMA, intraductal pancreatic intraepithelial adenoma; IPMC, intraductal papillary mucinous carcinoma; PanIN, pancreatic intraepithelial neoplasia; Pb, pancreatic body; PDAC; pancreatic ductal adenocarcinoma; Ph, pancreatic head; Pt, pancreatic tail; SPACE, serial pancreatic aspiration cytological examination.						

Our present study is a multicenter collaborative research project involving both community hospitals and university hospitals. Although various reports on SPACE have been published to date, most have come from single institutions, primarily advanced medical centers such as university hospitals 6 7 13 15 16 18 . In our study, diagnostic accuracy was consistently high across facilities, mostly exceeding 80%. One facility with a lower accuracy rate was likely influenced by the small number of cases. Reproducibility of SPACE across both academic and community hospitals highlights its high external validity. These findings support broader clinical applicability of SPACE beyond specialized centers.

The present study had several limitations, the first of which were its retrospective design and relatively small patient cohort, which may have introduced bias in patient selection and treatment outcomes. Lack of a unified protocol for SPACE, including variations in cytological examination frequency and ENPD tube size, also may have influenced our results. In patients who did not undergo surgery, the final diagnosis relied on SPACE results, laboratory investigations, and imaging findings with a minimum follow-up of 12 months, which may lead to misdiagnosis in the case of indolent lesions.

## Conclusions

In conclusion, for focal pancreatic duct stenosis, SPACE demonstrated its utility in obtaining pathological specimens and yielding improved diagnostic sensitivity and accuracy compared with single cytology alone. However, this procedure should be performed with caution due to the relatively high incidence of pancreatitis. Future research should focus on improving safety, particularly in reducing risk for pancreatitis, and conduct of prospective evaluations of the diagnostic capability of SPACE using a standardized protocol.

## Data availability statement

The outcomes of serial pancreatic juice aspiration cytologic examination used to support the findings of this study are not publicly available because they containing information that could compromise the privacy of research participants but they are available from the corresponding author upon reasonable request.
